# Recent Advances in Discovery of Lead Structures from Microbial Natural Products: Genomics- and Metabolomics-Guided Acceleration

**DOI:** 10.3390/molecules26092542

**Published:** 2021-04-27

**Authors:** Linda Sukmarini

**Affiliations:** Research Center for Biotechnology, Indonesian Institute of Sciences (LIPI), Cibinong, Bogor 16911, West Java, Indonesia; linda.sukmarini@lipi.go.id

**Keywords:** drug discovery, natural products, bioinformatics, genome mining, bioanalytics

## Abstract

Natural products (NPs) are evolutionarily optimized as drug-like molecules and remain the most consistently successful source of drugs and drug leads. They offer major opportunities for finding novel lead structures that are active against a broad spectrum of assay targets, particularly those from secondary metabolites of microbial origin. Due to traditional discovery approaches’ limitations relying on untargeted screening methods, there is a growing trend to employ unconventional secondary metabolomics techniques. Aided by the more in-depth understanding of different biosynthetic pathways and the technological advancement in analytical instrumentation, the development of new methodologies provides an alternative that can accelerate discoveries of new lead-structures of natural origin. This present mini-review briefly discusses selected examples regarding advancements in bioinformatics and genomics (focusing on genome mining and metagenomics approaches), as well as bioanalytics (mass-spectrometry) towards the microbial NPs-based drug discovery and development. The selected recent discoveries from 2015 to 2020 are featured herein.

## 1. Introduction

Natural products (NPs) originating from plants, animals, marine organisms, and particularly from microbial sources continue to inspire novel discoveries in chemistry [1], biology [2], and medicine [3]. They possess immense structural and chemical diversity with a wide variety of biological properties. Most pharmacologically relevant antimicrobial, antiviral, anti-inflammatory and analgesic, and antitumor agents and approved small molecule drugs have either been NPs, their derivatives, synthetic compounds with NP pharmacophore, or their synthetic mimics. Notably, more than half of the new small molecule drugs have been developed from microbial NPs over the past decades [4,5].

Current interest in the discovery of NPs, especially from microbial sources, is mostly due to the failure of synthetic libraries to generate the expected number of developmental drug candidates in the pharmaceutical industry during the past 20–30 years. Additionally, the emergence of clinically relevant pathogens becoming increasingly resistant to currently used anti-infectives, i.e., antibiotics, warrants the search for novel bioactive metabolites in the field of microbial NPs [5,6,7,8,9]. However, finding novel NPs has become more difficult as the rediscovery of known NPs is still an increasing challenge. A high rate of the discovery of NPs was yielded by classical methods that recover only a fraction or even none of the desired secondary metabolites. The sharp decline in discoveries arose with limitations of the traditional top-down screening approaches. Those approaches, including bioassay- and chemical signature-guided isolations, have largely been exhausted and may no longer be capable of delivering novel lead compounds [10].

In the search for alternative methods, advancements made in bioinformatics and chemical analysis might hold the key to lead a renaissance in the field of microbial NP discovery. The growing knowledge of different biosynthetic machinery, drug targets, and resistance mechanisms has served as a launch platform to a new era in the methodological approach for drug discovery [11,12]. Given the rising limitations imposed by uncultivable strains and silent gene clusters, the integrative approach of bottom-up targeted screening, employing advanced analytical methods and guided by bioinformatics analysis, provides a promising alternative for unlocking the microbial metabolomes on an unprecedented scale. This approach eventually leads to disclosing the potential of microbial NP discovery [13,14,15]. This mini-review highlights in particular some of the most recent advances in microbial NP discoveries as well as their discovery examples in the last five years achieved by the use of genomic and metabolomic approaches. In terms of this, a genomic strategy uncovers the large number of microbial clustered genes (biosynthetic gene clusters) that encode the proteins responsible for the biosynthesis of a new NP that is undetected under standard fermentation conditions, while a metabolomics method embraces the global measurement of small-molecule metabolites from a microbe.

## 2. Bioinformatics- and Genomics-Driven Discovery

Genomics and metagenomics (which has also been described as environmental genomics, relating to the genomic DNA from an environmental sample) revealed the remarkable biosynthetic potential of microbial NPs and their vast chemical inventory that can be prioritized and systematically mined for novel or new secondary metabolites with desirable bioactivities. The growing application of bioinformatics into a standard practice in discovery projects has varied approaches to identify novel lead structures [16]. Herein, advances in genomics-driven NPs discovery covering bioinformatics-guided identification of biosynthetic gene clusters (BGCs) in (meta)genomes are briefly highlighted. Additionally, the application of innovative technology in situ cultivation in novel compound discovery is also included. 

### 2.1. Genome Mining Approach

Fueled by the fast development of genome sequencing technologies, genome mining evolved during the last decades and is currently an essential part of drug discovery efforts. The genome mining approach detects and analyzes the BGCs of the chemical compounds automatically (computationally) and subsequently connects those genes to molecules. Furthermore, the significance of this approach associated with other techniques leading to drug discovery, especially of microbial NP origin, has been extensively described elsewhere [17,18,19,20]. Although the genome mining approach showcases the full biosynthetic potential of a strain, it is not very worthwhile without linking the predicted secondary metabolite BGCs to their product. Moreover, to take full advantage of NP diversity, BGCs must be prioritized by product novelty or function. BGCs hold the key information to understanding and predicting a specific or a group of related metabolites. By identifying open reading frames (ORFs) in a gene sequence, one can set the borders of the protein-encoding genes, and therein the protein sequence can be predicted through bioinformatics tools. As in some cases, bioinformatics can reveal BGCs with high similarities as a fast evaluation for target novelty; consequently, the time invested with computational work would save extensive resources and efforts only to re-isolate a previously described compound [15,20,21,22,23].

As mentioned above, progressions made in bioinformatics are mainly owed to advancements in genomics. Hence, the wealth of genomic information has led to the development of multiple bioinformatics-guided genome mining tools that examine this genomic data to detect and annotate potential BGCs automatically. Nevertheless, the realization of the full potentials of bioinformatics is bound to improvements in information algorithms towards knowledge of NP biosynthetic machinery (e.g., ribosomally synthesized and post-translationally modified peptides/RiPPs and non-ribosomal peptide synthase/NRPS, and polyketide synthase/PKS) [24,25,26,27]. Several widely used online platforms are still in active development, as listed in Table 1. Many of these selected tools have been extensively reviewed [21,28,29,30,31,32,33,34]. 

**Figure 1:**
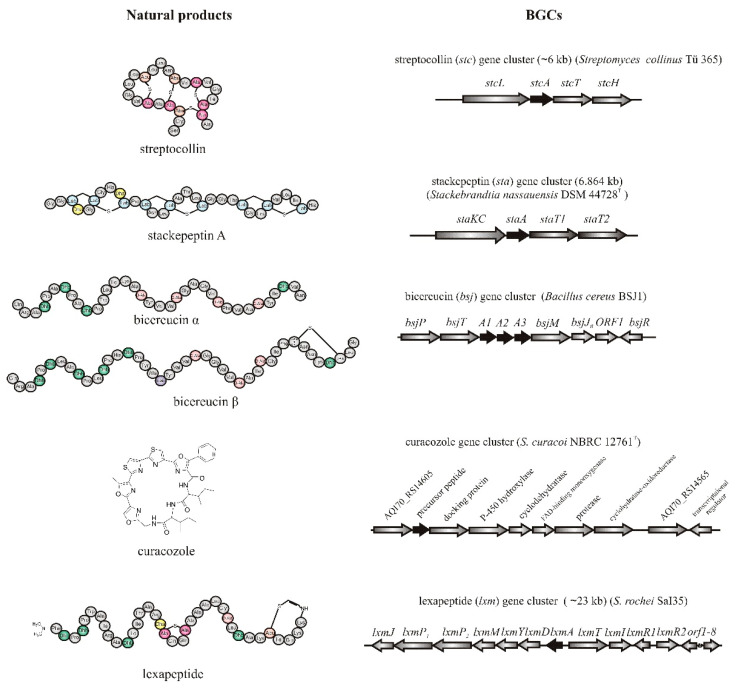
Selected recent examples of RiPPs and their BGCs discovering by the genome mining approach. The structural genes within BGC-encoded precursor peptides are depicted in black.

Streptocollin [46], stackepeptins [47], bicereucin [48], curacozole [49], and lexapeptide [50] (Figure 1) are the recent examples of RiPPs in which their BGCs were discovered using the genome mining approach. These metabolites were successfully characterized by heterologous expression and monitoring production in tandem mass spectrometry (MS) experiments. 

**Figure 2:**
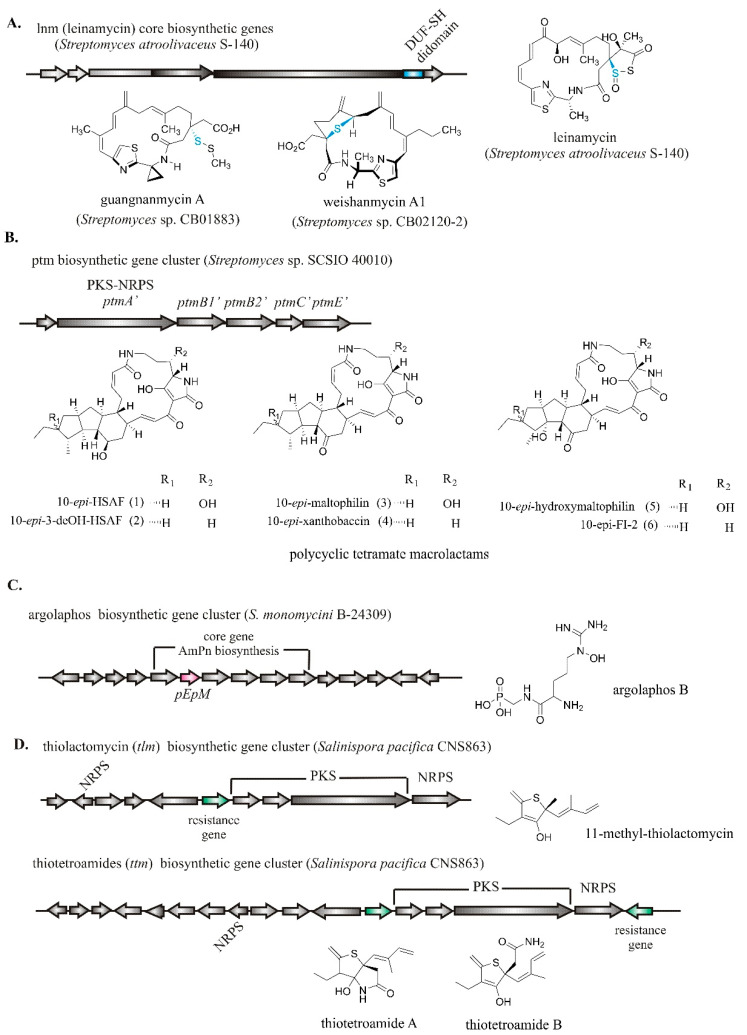
Selected recent examples of NRPS-PKS hybrids (**A**,**D**), polycyclic tetramate macrolactam (**B**), and phosphonate family of NPs (**C**) and their biosynthetic gene cluster discovery using the genome mining approach.

Moreover, two new NRPS-PKS hybrids, guangnanmycin A and weishanmycin A1, were discovered through BGC genome mining of promising anticancer drug leads leinamycin NP family (Figure 2A) [51], while five out of six new NRPS-PKS polycyclic tetramate macrolactams (Figure 2B) from the genome of *Streptomyces* sp. SCSIO 40,010 were identified to have cytotoxic activity [52]. In addition, a broad-spectrum antibacterial of rare sulfur-containing phosphate argolaphos B (Figure 2C) was discovered by mining the genomes of 10,000 actinomycetes [53]. Another example is the thiotetronic acid antibiotics of a new thiolactamycin analog (11-methyl-thiolactomycin) and thiotetroamides A and B (Figure 2D) discovered by a resistance-directed genome mining strategy [54]. By targeting BGCs with duplicated housekeeping genes that may encode protein targets, one is now able to infer the target of uncharacterized NP by analyzing BGC-associated self-resistance genes without prior knowledge of the structure.

### 2.2. Culture-Independent Strategies and Revolution in Metagenomics

It has been estimated that less than 1% of the bacteria present in most environmental samples are readily susceptible to cultivation using current fermentation technologies. Moreover, 5% of fungal species have been described, while many remain understudied despite their significant source of bioactive metabolites. Extensive studies of microbial 16S rRNA have revealed that the natural diversity of the prokaryotes by far exceed the number of bacteria that have been described to date. Therefore, in an attempt to decipher novel bioactive metabolites from unidentified microbes, researchers have explored several culture-independent approaches, including the current diffusion chamber technology, isolation chip (iChip). This multichannel device allows for the diffusion of nutrients and growth factors through the chambers. It enables the growth of uncultured bacteria in their natural environment. The application of this technology has led to the discovery of a novel depsipeptide antibiotic teixobactin from a previously unculturable β-proteobacteria named Eleftheria terrae. Interestingly, this antibiotic has displayed no detectable resistance thus far. The BGC identification using a homology search revealed that teixobactin consists of two large NRPS-encoding genes [55,56,57].

Moreover, the other culture-independent approach of metagenomics has also been established. Metagenomics relies on sampling environmental DNA (eDNA) and assessing their metabolomics independent from the producing organism. This has great implications when considering strains challenging to isolate or cultivate, such as strains from extreme environments and symbionts of marine organisms [16,58]. The revolution in this approach encompassing the phenotypic and homology DNA screening strategies in situ has been ameliorated by the advancement of next-generation sequencing (NGS) technologies [59,60,61]. By directly capturing eDNA from the environment and subsequently identifying, isolating, and expressing BGCs in a heterologous host, metagenomics has the potential to bring biosynthetic diversity from the environment into drug discovery pipelines.

**Figure 3:**
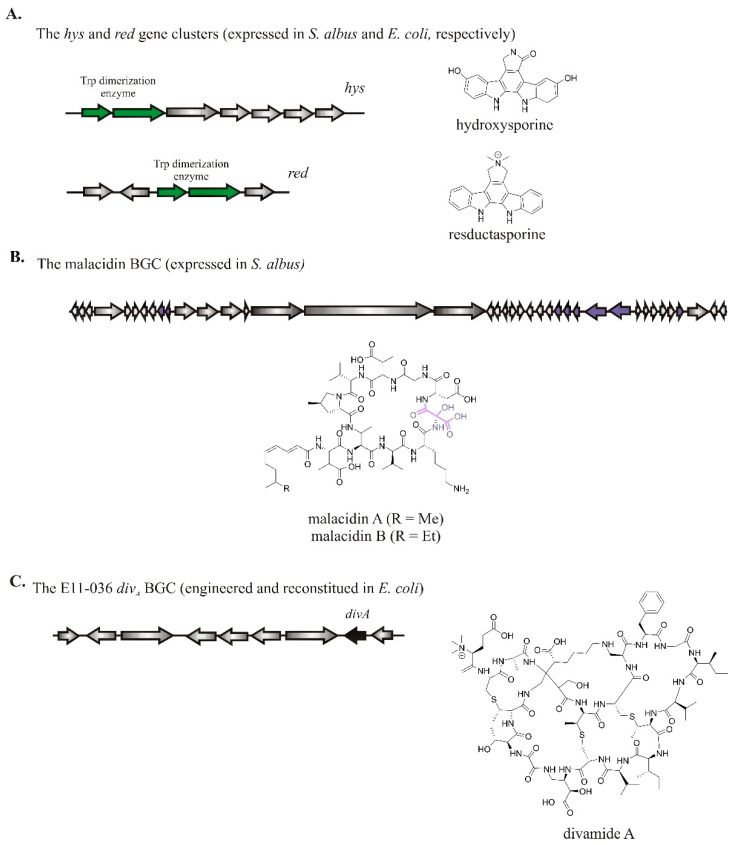
Selected recent examples of NP discovery employing the metagenomic approach. (**A**) The *hys* and *red* gene clusters containing Trp dimerization enzyme sequence tag (depicted in green), which were expressed in *S. albus* and *E. coli*, respectively, afforded two new rare tryptophan dimers, hydroxysporine and reductasporine. (**B**) The malacidin BGC containing the Asp4 (the domain responsible for incorporating the first aspartic acid) gene sequence that was expressed in *S. albus* led to the production of malacidins A and B containing a rare 3-hydroxyl aspartic acid moiety (HyAsp, highlighted in purple). (**C**) The metagenome sequencing of the DNA sample of whole-tunicate *D. molle* E11-036 revealed the BGC of *divamide A* (the core peptide divamide A is shown in black).

A study by Brady and his co-workers [62] employing targeted metagenomics of soil samples from different geographic regions led to the discovery of two new antifungal structures belonging to the rare class of tryptophan dimers NPs, hydroxysporine and reductasporine (Figure 3A). Soil samples were pre-screened to identify the most phylogenetically unique CPAS (responsible for the dimerization of activated Trp) gene sequences. Molecules associated with this gene were accessed through targeted metagenomic library construction and heterologous expression in *S. albus* or *E. coli*. Moreover, a class of calcium-dependent antibiotics called malacidins (Figure 3B) was recently discovered by the metagenomics approach of 2000 unique soils. These antibiotics exhibited activity against multidrug-resistant pathogens and sterilized methicillin-resistant *Staphylococcus aureus* [63]. The cyclic lipopeptides of malacidins A and B contain eight amino acids macrocycles and polyunsaturated lipid, incorporating a rare 3-hydroxyl aspartic acid. Another recent breakthrough discovery was the finding of antiviral peptide divamide A (Figure 3C) exhibiting activity against the human immune virus infection. These compounds were synthesized by symbiotic cyanobacteria *Prochloron didemni* living in marine tunicate *Didemnum molle* E11-036 [64].

## 3. Technological Advancements in Bioanalytics: Mass Spectrometry-Based Metabolomics

The key step in compound detection and identification relies directly on analytical instrumentation and data processing software for increased sensitivity and accuracy. Given the need for increased sensitivity in metabolomics, mass spectrometry (MS) is a predominant analytical technique with wide applicability in high-throughput screening programs. It has the potential to uncover elemental composition; structural information, i.e., mass-to-charge ratios (*m/z*); isotopic patterns; and abundance, as well as fragmentation patterns of molecules. Current separation techniques, including high-performance liquid chromatography (HPLC) or ultra-high-pressure liquid chromatography (UPLC), as well as gas chromatography (GC), are routinely coupled to MS towards efficient detectability of the generated ions. This coupled system has proved a powerful technique that has contributed towards metabolic profiling [65,66,67]. It has been known that chemical and electron impact ionization (EI/CI) frequently used with GC–MS and the more recent electrospray ionization (ESI) and matrix-assisted laser desorption/ionization (MALDI) allow for the analysis of complex molecules such as proteins and peptides. Moreover, the mass analyzer has been developed to employ various detectors, including the time-of-flight analyzer (TOF), the quadrupole ion trap (QIT), the ion cyclotron resonance (ICR), and the orbitrap. While the single-stage MS technique mainly reveals the mass compound, the fragmentation through collision-induced dissociation (CID) for tandem MS (MS/MS or MS^n^) and during electron ionization (EI) provides the building blocks used to characterize molecules and study their fragmentation behavior. The interpretation and in-depth analysis of these molecular fragments towards accurate identification of NP compounds have been made possible by recent MS technique advances [68,69,70,71].

**Figure 4:**
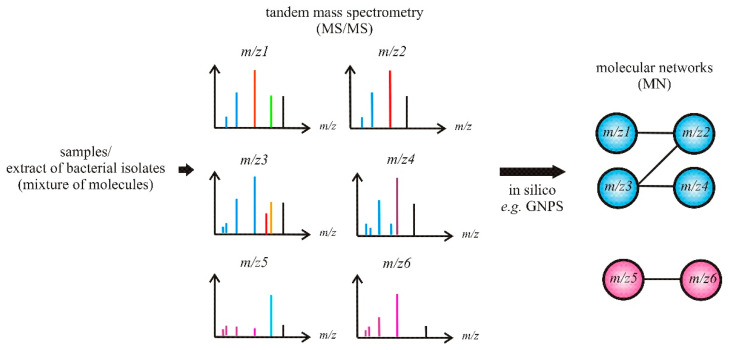
Schematic of molecular networking (MN)-based dereplication.

Recent advances in MS that integrate molecular networking (MN) of the MS/MS data have allowed for more rapid dereplication of known molecules from complex mixtures (Figure 4), which in turn have enabled not only the identification of related analogs but also contributed towards unraveling novel compounds by avoiding re-isolation of known compounds. It can be used to explore thousands to millions (and potentially billions) of MS/MS spectra without any prior knowledge regarding the chemical composition of samples. An open-access MN platform Global Natural Products Social Molecular Networking (GNPS; http://gnps.ucsd.edu, accessed on 10 February 2021), can automatically perform a spectral library search for known molecules (if available in public MS/MS spectral libraries) [72,73]. Furthermore, Allard et al. (2016) [74] integrated MN and an extensive in silico MS/MS database, offering a more powerful tool to navigate through the chemistry of complex NP extracts, dereplicate metabolites, and annotate analogs. 

**Figure 5:**
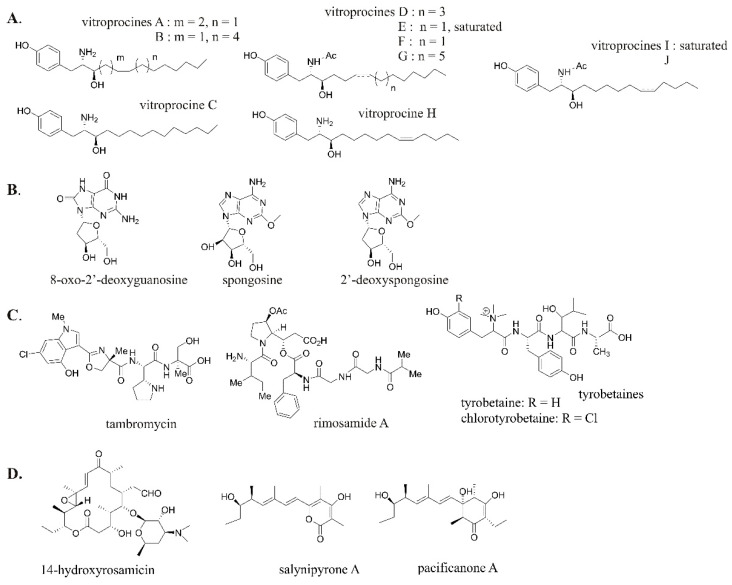
Selected recent examples of NPs discovered by the MN-based approach and metabologenomics. (**A**) Polyketides. (**B**) Sphongonucleosides. (**C**) NRP and the hybrid NRPS-PKS. (**D**) Glycosylated NPs.

Application of the MN approach to marine microbial *Vibrio* strains has led to the discovery of a series of antibacterial polyketide vitroprocines A-J (Figure 5A) [75] and anti-inflammatory and analgesic sphongonucleosides (Figure 5B) [76]. Recently, MN has been coupled with genome mining to dig more into the BGCs responsible for metabolite production. This method may also be applied in elucidating biosynthetic pathways and conjugation with stable-isotope labeling by amino acids in cell culture (SILAC) in order to provide more comprehensive insights into metabolomics studies, e.g., NPRS-PKS nidulin A [77] and colibactin [78,79]. The information provided by the MN–BGC correlation apparently can be exploited to augment discovery, isolation, and structural prediction of novel compounds produced by an organism, including a microbial strain [80,81]. An association between genomics and metabolomics data allowed for the detection of three new antibiotic NPs, columbamides A, B, and C [82], and a new type of thiomarinol [83] from marine bacteria. Additionally, MS-guided genome mining called metabologenomics detects new NPs and connects them with their BGCs. Matched BGC sequence information may be harnessed to elucidate compound structures further and/or to identify additional molecular features for searching. Metabolomics works by grouping similar BGCs from diverse bacteria into gene cluster families (GCFs) [16,84,85,86]. It should be noted that peptide-based NP discovery has primarily employed this method (peptidogenomics) due to its well-characterized biosynthetic machinery. Non-ribosomal peptide (NRP) tambromycin [87] and the hybrid NRPS-PKS rimosamides [88] are examples of novel NPs detected by the metabologenomic approach (Figure 5C). Through metabologenomic workflow of a 178-strain actinomycetes dataset applying scoring metrics to identify correlations between NP and GCF, these peptides were successfully afforded. Furthermore, a recent discovery of NRP tyrobetaines (Figure 5C) utilizing this workflow in combination with MN showed the great potential of MN-based metabologenomics for identifying novel NPs [89]. The approach has also been extended to the discovery of glycosylated NPs (glycogenomics) such as the marine-derived antibiotic rosamicin derivative and salinipyrone A and pacificanone A (Figure 5D) [90]. By matching tandem MS spectra of a marine bacterium *Salinispora pacifica* SNS237 with the BGC of type I PKS encoding desosamine (deoxysugar) biosynthesis, research revealed several rosamicin derivatives. Interestingly, mutagenesis experiments have revealed that salinipyrone and pacificanone seem to be by-products of the rosamicin PKS. Moreover, both peptidogenomic and glycogenomic approaches, as well as metabologenomics, have been extensively reviewed very recently elsewhere [81]. 

## 4. Conclusions

Remarkably, advancements in bioinformatics tools, genomics, and bioanalytics (particularly in MS) have recently enhanced the field of microbial NP research. These strategies outlined above offer alternatives to accelerate NP drug discovery over conventional methods efficiently. With continued significant progress in both genomics and metabolomics approaches and/or combined with synthetic biology, the microbial NPs discovery field shows strong signs of developing and is ready to lead at the forefront of delivering drugs or drug leads.

## Figures and Tables

**Table 1:** Selected latest bioinformatics tools dedicated to genome mining NPs (2015–2020).

Platform	Description	Web Server URL	Reference
BIG-FAM	Global biosynthetic space of microbial BGC families database	https://bigfam.bioinformatics.nl, accessed on 10 February 2021	Kautsar et al. [35]
MIBiG 2.0	Minimum information on biosynthetic gene clusters (MIBiG) standard respiratory of characterized BGCs	https://mibig.secondarymetabolites.org, accessed on 10 February 2021	Kautsar et al. [36]
antiSMASH 5.0	Automated pipeline to mine secondary metabolite BGCs	https://antismash.secondarymetabolites.org, accessed on 10 February 2021	Blin K et al. [37]
PRISM 4	Automated pipeline to mine secondary metabolite BGCs	http://prism.adapsyn.com, accessed on 10 February 2021	Skinnider et al. [38]
BAGEL 4	Mining of RiPP and bacteriocins BGCs	http://bagel4.molgenrug.nl, accessed on 10 February 2021	Van Heel et al. [39]
BiG-SPACE - CORASON	Biosynthetic gene similarity clustering and prospecting engine	https://bigscape-corason.secondarymetabolites.org, accessed on 10 February 2021	Navarro-Muňoz et al. [40]
ARTS	Mining of BGCs on the basis of the prediction of antimicrobial resistance genes that are part of BGCs	https://arts.ziemertlab.com, accessed on 10 February 2021	Alanjary et al. [41]
CASSIS/SMIPS	Mine for PKS, NRPS, and DMATS anchor genes (SMIPS) in fungal genomes; predict gene clusters around anchor genes on the basis of conserved promoter regions	https://sbi.hki-jena.de/cassis/, accessed on 10 February 2021	Wolf et al. [42]
IMG-ABC	A comprehensive database of secondary metabolite BGCs	https://img.jgi.doe.gov/abc, accessed on 10 February 2021	Hadjithomas et al. [43]
RiPPMinner	Analysis of RiPP precursor peptides to predict structural features	https://www.nii.ac.in/~priyesh/lantipepDB/newpredictions/index.php, accessed on 10 February 2021	Agrawal et al. [44]
RiPP-RODEO	Mining and analysis of RiPPs	https://www.ripprodeo.org/, accessed on 10 February 2021	Tiez et al. [45]

## Data Availability

No new data were created or analyzed in this study. Data sharing is not applicable to this article.
